# A Dream Realized

**Published:** 1888-07

**Authors:** 


### A DREAM REALIZED.

"Speaking about dreams, I can give you a true story of one I had once that left a lasting impression upon my mind," said David Whelton, the foreman of the Boston & Maine roundhouse, as a *Globe* reporter sat in the cosy office at Somerville the other night. "And I had rather give it to the *Globe* than any other paper,"said Mr. Whelton, "for I'm a workingman, and the *Globe* stands up for our interests every time. But the dream was as follows :

"Thirty-five years ago I was working for the Boston & Maine on the Reading section. The section foreman, with whom I boarded was my uncle, Tim Canty, who holds the same position now. There were two other men in the gang, one of whom, Patrick Burns, is still alive in Reading, and will join with my uncle in vouching for the truth of my story. One night, after finishing my day's work, I retired as usual, and in my sleep had the following dream : I though I had arisen the following morning, and while we were going to the place where we were to make some repairs in the track, on a curve about a mile and a half beyond Reading, we came upon a passenger train in the ditch. I remember how vividly the whole scene came before me, and how we at once went to work digging the dead and wounded out of the wreck and piling them on the hand-car and carrying them to the village.

"On awaking I arose and told my aunt what an impression the dream had made upon me. She laughed and told me it was unlucky to tell a dream before breakfast. I then went into the yard and told it to my uncle, and on going to work I told it to the other section hands and expressed my opinion that the dream was a presentiment of evil, but they pooh-poohed at it until we came to the curve I saw in my dream, when, on rounding it, what was our surprise to find a wreck just as I had dreamed of.

"A passenger train, drawn by the engine Hickley, No. 19, with Joseph Langley, engineer, and Daniel Smart, conductor, lay in the ditch. The train consisted of four passenger cars and a baggage car. There were no smoking cars in those days, and the baggage car was filled with laborers going to their work. This car was turned completely over, and the heavy flooring and trucks had pinned many of the men down, killing some and injuring others severely. I at once went back for doctors, and the rest went to work digging out the injured and piling them on the hand-car, exactly as in my dream.

"The immediate fulfilling of my dream caused a great sensation, and, as I had related it in detail to a number before we went to work, no doubt could be raised in the minds of any as to the truth of the story.

Mr. Whelton has been at work on the Boston & Maine for about thirty-five years, with the exception of about two years during the war, when he was in the cavalry service. He has charge of over fifty engines and oversees about a dozen men. He is one of the most trusted men on the road, and all who come in contact with him testify as to the confidence which can be placed in his veracity. This dream had made such an impression on him that he is a firm believer in the reliability of dreams in general. He can be seen any evening at the roundhouse, where he and Mr. Higgins, who has general charge of affairs, show every courtesy to visitors.—*Boston Globe.*

